# Dental Anxiety Before Non-Surgical Periodontal Treatment: Evaluating the Interaction of Gender and Education Level

**DOI:** 10.3290/j.ohpd.c_2800

**Published:** 2026-09-09

**Authors:** Tüba Bayat, Koray Cömert

**Affiliations:** a Tüba Bayat Assistant Professor, Sakarya University Faculty of Dentistry, Department of Periodontology, Sakarya, Turkey. Conceptualization, data collection, formal analysis, wrote original draft, reviewed and edited the manuscript.; b Koray Cömert Research Assistant, Sakarya University Faculty of Dentistry, Department of Periodontology, Sakarya, Turkey. Data collection, wrote original draft, reviewed and edited the manuscript.

**Keywords:** dental anxiety, education, gender, interaction, periodontal therapy.

## Abstract

**Purpose:**

Dental anxiety is a multidimensional factor that influences treatment compliance and periodontal outcomes. This investigation evaluated how gender, educational level, and their combined interaction relate to dental anxiety in a sample of patients receiving initial non-surgical periodontal therapy (Phase I), minimizing potential confounding effects from procedural differences.

**Materials and Methods:**

This cross-sectional study included 200 volunteers (100 females, 100 males) who received Phase I periodontal therapy. The required sample size was estimated using G*Power analysis to achieve a statistical power of 95%. Dental anxiety was assessed using the Modified Dental Anxiety Scale (MDAS). Age homogeneity between genders was confirmed using the independent samples t-test. Data were analyzed using two-way ANOVA, the Games–Howell post-hoc test, and simple effects analysis. Effect sizes were determined via partial eta squared (partial η²).

**Results:**

No statistically significant difference in mean age was found between genders (p = 0.890). Two-way ANOVA revealed no statistically significant main effect for gender (p = 0.683) or educational level (p = 0.315). However, a statistically significant interaction effect with a large effect size was observed between gender and educational level (F(3,192) = 13.237; p < 0.001; partial η^2^= 0.171). Simple effects analysis demonstrated that anxiety levels statistically significantly decreased as educational level increased in females (partial η² = 0.197), whereas in males, anxiety levels paradoxically increased with higher education (partial η² = 0.175).

**Conclusion:**

The impact of education on dental anxiety is not uniform but is statistically significantly modulated by gender. These findings emphasize the necessity of moving beyond standardized communication toward a customized, gender- and education-tailored chairside approach to optimize compliance during non-surgical periodontal therapy.

Dental anxiety is a frequently encountered clinical condition that negatively influences oral hygiene practices, treatment adherence, and overall clinical outcomes. During periodontal therapy, invasive procedures, anticipated pain, and prior negative dental experiences can significantly elevate anxiety levels. Long-standing clinical evidence suggests that individuals with heightened dental apprehension often tend to postpone professional interventions—a behavior that subsequently accelerates the progression of periodontal tissue deterioration.^1,13,17^ In this context, initial nonsurgical intervention remains the foundational element of periodontitis care, establishing the primary baseline for halting disease progression and ensuring prolonged clinical and peri-implant stability.^19,22,26^ Crucially, proactively managing patient anxiety at this entry point is paramount; severe apprehension can compromise long-term compliance, which thus necessitates more invasive secondary interventions that could have otherwise been avoided. Aside from its direct therapeutic merits, the successful control of periodontal diseases mitigates substantial economic and healthcare burdens, emphasizing the critical value of timely and cost-effective clinical protocols.^16,18^

Consequently, identifying the determinants that shape anxiety prior to periodontal interventions is critical for optimizing preventive and therapeutic patient management strategies. The Modified Dental Anxiety Scale (MDAS), widely used in clinical evaluations, is a standardized instrument with proven validity and reliability across various populations, including Turkish cohorts.^10,11^ Crucially, individual psychological profiles and general oral health perceptions can directly modulate patient compliance during this primary phase of care.^9^

Extensive research has focused on the sociodemographic determinants of dental anxiety, with a recurring consensus that women bear a greater psychological burden in clinical settings than men.^2,15,24^ In contrast, the relationship between educational attainment and dental anxiety exhibits a more varied and contradictory pattern. While some studies suggest that higher education may reduce anxiety by improving health literacy, others fail to confirm this association or report conflicting findings.^2,3,5^ These inconsistencies suggest that evaluating education as an isolated variable may be insufficient, and that its impact on dental anxiety may be modulated by other demographic factors.

Most studies to date have focused on the independent effects of sociodemographic variables, largely overlooking the dynamic interactions between these factors. Specifically, the potential interaction between gender and educational level remains an underexplored area in periodontal research. Addressing the hypothesis that the effect of education on dental anxiety may differ according to gender represents a critical gap in understanding the psychological profiles of patients with periodontal disease. Examining these interactions may support the design of individualized, evidence-based patient management strategies, allowing clinicians to move beyond uniform, generalized approaches.^3,15,21^

The primary objective of this investigation was to evaluate how gender, educational level, and their combined interaction relate to dental anxiety specifically within a sample of patients receiving initial non-surgical periodontal care (Phase I), thereby minimizing potential confounding effects arising from procedural differences. By exploring these demographic dynamics, this research aims to provide practical insights that help clinicians recognize variations in patient anxiety and refine their routine therapeutic communication strategies.

## MATERIALS AND METHODS

### Study Design and Ethical Approval

The present cross-sectional investigation was conducted in the Periodontology Department at the Faculty of Dentistry, Sakarya University. The study population comprised patients indicated for Phase I non-surgical periodontal treatment. Ethical clearance was officially granted by the Scientific Research Ethics Committee of the Sakarya University Faculty of Health Sciences (Decision No. 275; Date: 16.02.2026). Prior to enrollment, all volunteers were briefed on the study’s nature, and formal written consent was secured from each individual. The entire research process adhered to the ethical standards established in the Declaration of Helsinki.^27^

### Sample Size and Participants

To ensure robust statistical validity, the required sample size was estimated via G*Power software (v. 3.1.9.7, Heinrich Heine University; Düsseldorf, Germany). Using a two-way ANOVA framework with a medium effect size (f = 0.25), a 5% alpha level, and a target power of 0.95, the analysis indicated a minimum of 172 subjects. To bolster this power and compensate for potential dropouts, 200 consecutive volunteers (balanced by gender: 100 females, 100 males) were recruited.

Eligibility was defined by being at least 18 years old, literate, and in need of initial periodontal therapy. Conversely, patients were excluded if they were unable to provide consent, had a diagnosed psychiatric history, were currently using sedatives or anxiolytics, or presented with acute dental pain necessitating emergency care.

### Data Collection

Psychometric evaluation of dental anxiety was conducted using the Modified Dental Anxiety Scale (MDAS).^10^ This five-item tool assesses various clinical scenarios on a 5-point Likert scale, yielding a total score between 5 and 25; elevated scores reflect heightened apprehension. The Turkish adaptation of the MDAS, previously validated for reliability,^11^ was employed. Participants completed the questionnaire in a controlled, quiet environment before any clinical procedures.

Gender and educational attainment were designated as the primary independent variables. Educational attainment was classified into four categories: primary education, high school, university, and postgraduate (master’s/doctoral) education.^20^

### Statistical Analysis

Statistical analyses were performed using SPSS (version 26.0, IBM; Armonk, NY, USA). Continuous variables were expressed as mean ± standard deviation, while categorical variables were presented as frequencies (n) and percentages (%). Data normality was assessed using the Shapiro–Wilk test and visual methods (histograms and Q–Q plots). Homogeneity of variances was evaluated using Levene’s test.

An independent-samples t-test was used to confirm age homogeneity between gender groups. Two-way ANOVA was applied to evaluate the main effects of gender and educational level, as well as their interaction, on dental anxiety scores. Due to the violation of homogeneity of variances (p < 0.05), the Games–Howell post-hoc test was used for multiple comparisons. Simple effects analysis was conducted to further examine the source of the interaction. Effect sizes were reported as partial eta squared (partial η²). A p-value < 0.05 was considered statistically significant.

## RESULTS

### Demographic Characteristics of the Participants

The comparison of mean ages of the participants according to gender is presented in Table 1. The analysis revealed no statistically significant difference between female and male participants in terms of mean age (p = 0.89), indicating that the sample was homogeneous with respect to this key demographic variable.

**Table 1:** Comparison of mean age of participants by gender

n (%)	Age	Mean ± SD	Min-Max	p
**Gender**				
Female	100 (50%)	41.7 ± 14.3	18-75	0.890
Male	100 (50%)	42.1 ± 15.7	18-77	
Total	200 (100%)	41.9 ± 15.0	18-77	
Statistically significant at p<0.05 (independent samples t-test); n: number of participants; SD: standard deviation; min-max: minimum and maximum values.				

### Analysis of Dental Anxiety (MDAS) Scores

Table 2 summarizes the collective influence of gender and educational level on dental anxiety measurements. Two-way ANOVA revealed that neither gender nor educational level exerted a statistically significant independent main effect on anxiety scores (p > 0.05). Conversely, a robust and statistically significant interaction was identified between these two variables, statistically significantly shaping the observed dental anxiety profiles (p < 0.001; partial η² = 0.171).

**Table 2:** Two-way ANOVA results for the effects of gender and educational level on MDAS scores

Source	DF	F	p	η^2^p
Gender	1	0.167	0.683	0.001
Education	3	1.190	0.315	0.018
Gender x education	3	13.237	0.001*	0.171
Statistically significant at p<0.05; DF: degrees of freedom; F: F-statistic; η^2^p: partial eta squared.				

**Fig 1:**
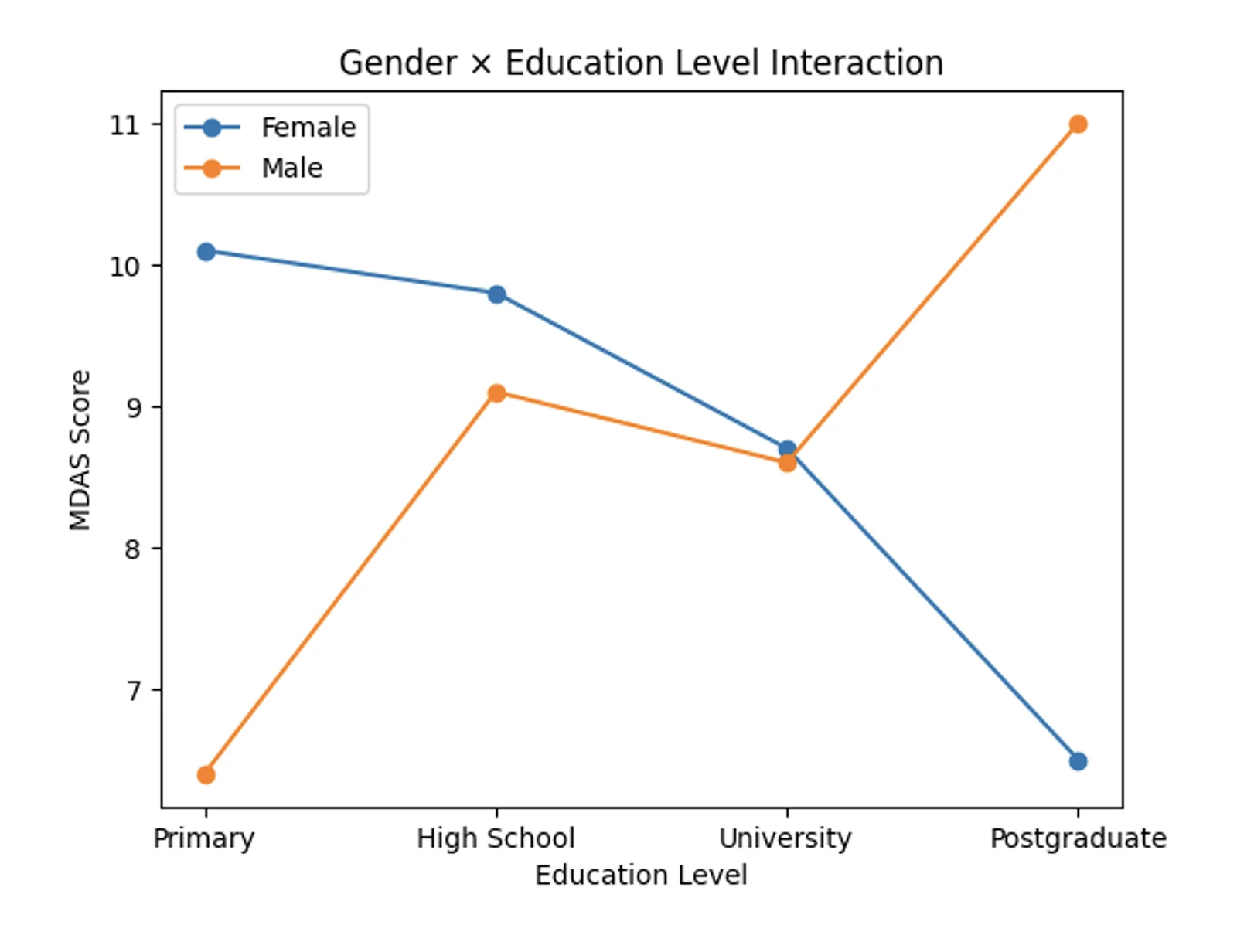
Variation in mean MDAS scores according to the ınteraction between gender and educational level.

Figure 1 shows that as educational level increases, a marked decrease in anxiety scores is observed among females, whereas in males, anxiety scores increase with higher educational levels, indicating a crossover interaction. To identify the specific group differences underlying this interaction, the results of the simple effects analysis and the Games–Howell post-hoc test are summarized in Table 3.

**Table 3:** Comparison of MDAS Scores by educational level within gender groups

Gender	Educational level	n	Mean ± SD	F	p	η^2^p	Significant difference (Games–Howell)
Female	Primary	25	10.1 ± 3.1	7.87	0.001*	0.197	Postgraduate < primary
	High school	25	9.8 ± 2.7				Postgraduate < high school
	University	25	8.7 ± 3.2				Postgraduate < university
	Postgraduate	25	6.5 ± 2.6				
Male	Primary	25	6.4 ± 1.4	6.77	0.001*	0.175	
	High school	25	9.1 ± 3.3				Primary < High School
	University	25	8.6 ± 3.4				Primary < University
	Postgraduate	25	10.1 ± 3.1				Primary < Postgraduate
Statistically significant at p<0.05; DF: degrees of freedom; F: F-statistic; η^2^p: partial eta squared.							

According to the results presented in Table 3, educational level had a statistically significant effect on anxiety scores among female participants (p < 0.001). Games–Howell analysis indicated that females with a postgraduate education had statistically significantly lower anxiety scores compared to all other educational groups (p < 0.05). A statistically significant effect of educational level was also observed among male participants (p < 0.001). However, in contrast to females, males with primary education exhibited statistically significantly lower anxiety scores than those with high school, university, and postgraduate education (p < 0.05).

Simple effects analysis revealed that the impact of educational level on dental anxiety demonstrated a large effect size in both females (partial η² = 0.197) and males (partial η² = 0.175). These findings confirm that educational level is a strong and fundamental determinant of variations in dental anxiety scores in both genders.

## DISCUSSION

This research primarily demonstrates that, although gender and educational level do not exhibit statistically significant independent main effects on dental anxiety, their interaction exerts a substantial influence on MDAS scores. Our results indicate that the effect of education on dental anxiety follows a contrasting trajectory based on gender: anxiety levels decrease as educational attainment increases in females, whereas a paradoxical increase is observed in males. The large effect size of this interaction (partial η² = 0.171), based on Cohen’s criteria, suggests a meaningful clinical relevance for this dynamic.^8^

The widespread occurrence of dental anxiety and its links with sociodemographic variables are extensively documented in the literature.^12,14,24^ However, the absence of statistically significant main effects for gender and education in the present sample is noteworthy. Although a broad consensus in the literature reports higher dental anxiety in females,^2,15,24^ our findings suggest that this discrepancy is modulated by educational attainment. Furthermore, the lack of a statistically significant difference in mean age between gender groups (p = 0.890) suggests that the observed anxiety patterns operate independently of chronological age.^23,25^

Within the biopsychosocial framework, this interaction could potentially be explained by distinct underlying psychological mechanisms. The rising anxiety scores among highly educated males (partial η² = 0.175) might be linked to a higher need for cognitive predictability and situational control in clinical environments. In general psychological literature, it is suggested that a passive patient role during clinical interventions can conflict with expectations of personal control.^1,21^ However, since the present study did not utilize specific psychological scales to directly quantify baseline control constructs or individual personality dimensions, this interpretation remains a theoretical hypothesis rather than an established causal link for this specific subpopulation. Conversely, the downward trend in anxiety among highly educated females (partial η² = 0.197) could be attributed to advanced oral health literacy, which typically mitigates uncertainty regarding dental interventions.^4^ This perspective aligns with research previously published in this journal, which emphasizes that health awareness and educational background are critical determinants of dental anxiety profiles across diverse demographics.^7^ Increased knowledge renders the treatment process more predictable, appearing to be a fundamental factor in mitigating anxiety among female patients.^6,10^

Furthermore, the seemingly paradoxical increase in anxiety observed among highly educated males may reflect a more reflective engagement with prior negative dental experiences. Individuals with higher educational attainment might be more inclined to cognitively process and internalise these experiences, thereby amplifying their emotional impact. In this context, a history of traumatic dental encounters remains a well-established and influential predictor of anxiety.^11^ It is possible that individuals with higher education engage more deeply with their past clinical exposures, at times revisiting and reinterpreting negative dental events in ways that sustain or intensify current apprehension. Taken together, these findings indicate that clinician-patient communication and anxiety management should adapt to more flexible, patient-centric communication frameworks prior to periodontal therapy. Considering the interplay between gender and educational level can facilitate more effective chairside interventions and improve long-term treatment compliance.

This study was subject to several limitations. Crucially, the cross-sectional design of this investigation precludes the tracking of longitudinal fluctuations in dental anxiety; therefore, the identified associations within this specific patient population should not be interpreted as definitive causal pathways. Additionally, relying on self-reported survey instruments may introduce potential response bias, as participants might downplay their genuine levels of dental apprehension due to social desirability. Furthermore, while procedural variability was minimized by focusing strictly on initial nonsurgical periodontal therapy, individual pain thresholds and detailed histories of past traumatic dental encounters—both recognized as strong predictors of anxiety, were not formally quantified. Finally, because this study was conducted within a single-center regional sample, cultural variations might influence these interactive dynamics, which may limit the direct generalizability of the findings to different global settings. Despite these limitations, the high statistical power and large effect sizes demonstrate that the interaction between gender and education remains a critical determinant of dental anxiety profiles in periodontal clinical practice.

## CONCLUSION

The current study demonstrates that the effect of educational level on dental anxiety is not uniform but is statistically significantly modulated by gender, showing contrasting trends in males and females. While the independent main effects were not statistically significant, the strong interaction effect indicates that dental anxiety is shaped by the combined influence of these demographic factors rather than isolated variables. Specifically, the paradoxical increase in anxiety among highly educated males—potentially linked to altered control dynamics—and the decrease in anxiety among highly educated females due to improved health literacy appear to be critical drivers of the psychological profiles of periodontal patients. From a clinical perspective, identifying these interaction patterns is essential for developing individualized anxiety management strategies prior to initial periodontal interventions. Tailoring chairside communication and psychological preparation to address the specific needs of these patient profiles during nonsurgical periodontal therapy can enhance treatment compliance and patient engagement. Ultimately, recognizing this gender-education interplay may support treatment continuity, facilitate the maintenance of periodontal health, and improve long-term clinical outcomes.
